# An Old Army Surgeon

**Published:** 1889-03-02

**Authors:** 


### An Old Army Surgeon.

Although medical men have been attached to armies from very early times, the foundation of modern military surgery may be said to have been been laid by Ambroise Paré, a Frenchman, whose discoveries cast an Æsculapian glory over the sanguinary sixteenth century, and who certainly ought, if he only knew it, to be numbered among the tutelary saints of Tommy Atkins.

Ambroise Paré was born at Laval in 1509, and was in due course sent to study in the College of Surgeons of his time—that is, he was apprenticed to a barber. It was not till after he had considerable experience of the practical side of his art, that he took up the scientific aspect of it. Indeed, it says something for the unassuming nature of the man, that in 1536, when he had already made a discovery that revolutionized surgical treatment, he should have thought it necessary to enter the Hôtel Dieu, at Paris, and there study for the modest degree of barber-surgeon. Indeed, vanity and over-confidence were never among Paré's faults; he was not infected with the modern mania for experiment ; and his first great discovery was made by accident—one of those accidents, however, that only occur to men of genius.

He was only nineteen when he began practice, following the French army to Italy as surgeon to Marshal Montéjean, colonel-general of the Infantry. At first he simply followed the treatment practiced by his seniors, and poured boiling oil into gunshot wounds, according to the tradition and habit of the time. On one occasion, however, the supply of boiling oil ran short, a number of wounded men could not be cauterised, and Paré made a salve of beaten egg, turpentine, and water, which he applied to the injuries, meaning to employ the orthodox treatment next day. But next day the soldiers who had been cauterised were feverish, and their wounds inflamed, while those to whom Paré had applied his new salve were better; and from that day the old barbarous treatment of gunshot wounds, inaugurated by Jean de Vigo, was condemned for ever.

Yet it was after he had thus revolutionised surgical practice that Paré became a student at the Hôtel Dieu. The absolute modesty of the man was shown in another way. Having heard of an Italian surgeon who was skilled in treating gunshot wounds he became his pupil, and accepted with reverence a prescription of the Italians which was really only a variation of his own. It consisted in adding to the other ingredients oil in which two young dogs had been boiled—alive, of course, for mediævalism was not yet past. Henceforth Paré lauded the virtues of the "oil of whelps," and was so much a man of his time, that, in spite of his own experience of the value of simple cures, he thought the dying screams of these hapless puppies gave an occult merit to the olive oil.

In most respects, however, he was a man who led his time. In 1528 he ventured to perform amputation at the elbow joint, and in 1543 in the Spanish campaign, which was one of Francis I.'s magnificent failures, he conceived the idea of facilitating the extraction of bullets by placing the wounded man in the posture in which he received the wound, thus giving the surgeon, if he were a thoughtful man, some idea of the course it had taken, and saving much unnecessary and harmful probing.

In 1558 he made his second great discovery. Hitherto the red-hot cautery had been the only means employed to stop the hemorrhage following amputation. Paré invented the method of tying the severed vessels—a discovery of the first importance in surgery. Subsequently he devoted his attention to the prevention of pyæmia and hospital gangrene, which he strove to combat by the application of alcohol and turpentine. He recognised the fact that these diseases were caused by impurities in the air and by the putrefaction of the tissues, and therefore condemned the application of various organic balms recommended by Bérenger de Carpo and Paracelsus, which were apt to infect the wounds.

When he had attained the height of his fame Paré received the degree of Master in Surgery from the College of Surgeons of Paris, and was appointed in succession first surgeon and councillor to King Henry III. He died on December 20th, 1590.

